# Ill. Charitable Eye and Ear Infirmary

**Published:** 1875-02

**Authors:** E. L. Holmes


### Clinics.

### ILLINOIS CHARITABLE EYE AND EAR INFIRMARY.

*Clinical Lectures on Diseases of the Eye,*

By E. L. HOLMES, M.D.

### SYMPATHETIC OPHTHALMITIS.

*Gentlemen:* You have the opportunity to-day of examining two cases, illustrating the history of a most important disease—a history comprising one of the saddest chapters in ophthalmic surgery: for the danger in many of these cases is extreme, although the symptoms are often so trifling and insidious as to excite no suspicion of an impending calamity in the minds of the patient or of the inexperienced practitioner.

The disease is usually termed sympathetic ophthalmitis. Its history in general is briefly this: a patient receives a wound in one eye in some one of the following ways; a fragment of metal or other substance has passed through the walls of the eye and become lodged in the intra-ocular tissues; a puncture has occurred near the union of the sclerotic and cornea; or there has been some serious injury of the globe, causing inflammation of the iris, ciliary bodies, and choroid. Less frequently, perhaps, the patient suffers with inflammation caused by a dislocated lens, or with chronic irido-choroiditis, with calcareous degeneration of the lens or choroid.

If the abnormal process in any of the above described diseases terminated in the eye originally injured and did not affect the sound one, there would be comparatively little harm, for a patient with only one perfect eye, though unfortunate, is many fold more fortunate than one who has lost both eyes.

Unhappily, as you observe in both these cases, the *unaffected eye* too often becomes the seat of a sympathetic inflammation of the choroid and iris, which can almost never be relieved, if it once commences, before vision is partially or totally destroyed.

Your future experience will undoubtedly teach you that the presence of a foreign body, as a fragment of percussion cap or steel, is the most frequent cause of danger. In regard to this class of injuries, a few points are worthy of notice. In ordinary cases, the removal of a foreign body from the vitreous humor is impossible, for hemorrhage, or, if the case has been neglected, the products of inflammation, conceal it from view. Even where the pupil is readily dilated and the object easily observed by the ophthalmoscope, the removal can rarely be accomplished, and in exceptional cases only by the most skillful use of instruments.

A foreign substance in the iris or lens may be removed with the surrounding portion of iris by iridectomy, or by the extraction of the lens as in the operation for cataract.

A minute fragment of heavy substance, thrown with sufficient force against the globe to make a puncture in its walls, has almost certainly passed into the intra-ocular tissues. Such a body often leaves an opening apparently more minute than the body itself.

You should remember, however, that a minute puncture may be made by a delicate point of a large body; but in such a case the piece is usually found after it rebounded from the eye.

The sensations of the patient are generally of no value in establishing a diagnosis whether there is or is not a foreign body in the globe.

From what has been said you are aware that an irido-choroiditis, however produced, is the disease predisposing to a sympathetic action in the other eye. The danger to the other eye is especially great when there is a persistent tenderness on gentle pressure over the region of the ciliary processes of the eye primarily affected.

The sympathetic inflammation is caused by an irritation of the ciliary nerves of the sound eye, induced by the diseased condition of the corresponding nerves in the injured one; and yet it is a fact, that, after the extirpation of an eye, amaurotic symptoms are occasionally observed in the remaining eye, in consequence of irritation of the end of the optic nerve by an ill-fitting artificial eye or from a constriction of the nerve in cicatricial tissue.

The symptoms of sympathetic ophthalmitis are, photophobia, dimness of vision, lachrymation, and especially an immovable and irregular pupil, the iris becoming attached to the anterior surface of the lens. As there is often but little pain, and as the symptoms develop very gradually, danger is often unsuspected. The symptoms may appear in three or four weeks. I have known a patient who lost a remaining sound eye after suffering periodic attacks of pain and inflammation in an injured eye for fifteen years.

In regard to treatment, there is comparatively little to be said, but that little is of vast importance. In every case where a foreign body in the globe cannot be extracted, the eye should be extirpated without delay. The same operation should be performed in all cases of atrophied globe, where there is an obstinate, long continued inflammation with neuralgia, and tenderness on pressure, which the ordinary remedies for irido-choroiditis have failed to relieve. It is of great importance that the patient should carefully protect the eye from the influence of strong light, dust, use of the eye in reading or writing, for this exposure tends to hasten the sympathetic inflammation.

Let us now turn to the history of the two cases before you.

Mr. ——, æt. 27, a mechanic, tossed a hatchet upon the floor. A very minute fragment from the edge of the tool was broken off and flew into the left eye. A couple of days after the accident, I made an examination, and found a minute opening in the sclerotic near the corneal border, blood in the anterior chamber to such an extent that the portions behind the pupil could not be seen. I expressed the opinion that the piece of metal was still in the globe, and urged the immediate extirpation of the latter. The patient, however, preferred to return home, some distance from Chicago, and await developments, although I described the possible dangers of the delay.

In about seven weeks he returned almost blind in the other eye from a severe irido-choroiditis. The patient based his reason for this delay on the fact that his friends had mentioned a case, in which a piece of steel had remained eleven years in an eye without serious consequences to the other eye. The globe was extirpated nearly eight weeks after the accident, too late, however, to modify the inflammatory action in the other eye. The piece of steel was found in the ciliary muscle. In this case you observe a glaucomatous process in the remaining eye, the great tension (hardness) of the globe being readily felt by gentle palpation with the tips of the two fore fingers. The disease has not yielded to iridectomy nor the use of pot. iod. and mercurials. The prognosis is certainly most grave.

In the other case before you, we have the following history: The young man, 20 years of age, received a piece of steel in the right eye a year ago last December. It was removed from the eye by his family physician, at the end of about seven weeks, the walls of the globe being left. Unfortunately the other eye became inflamed and gradually almost blind. At present we find the injured eye atrophied, without inflammation. The other is also without inflammation, but is partially atrophied, the tension being much less than normal. There is a cataractous lens with iris very extensively attached, and still the patient can see to go alone in the street, although with difficulty. As there is scarcely a hope that iridectomy would improve vision, and as the sight is slightly improving under the use of iodide of potash and atropine, we will, for the present, follow no other treatment.*

I cannot close these remarks without urging you to make this chapter of ophthalmology a subject of special study.

* I would insist upon the operation of iridectomy, if the iris was even slightly raised above the surface of the lens. It is, however, atrophied, and undoubtedly attached to the lens throughout its whole posterior surface.

You will at times be called upon to exercise your utmost prudence. Just as the question whether you should amputate a limb or attempt to save it, may be a very difficult one to solve at the proper time, so will be the question whether you should extirpate an eye or attempt to save it.

